# Do walking strategies to increase physical activity reduce reported sitting in workplaces: a randomized control trial

**DOI:** 10.1186/1479-5868-6-43

**Published:** 2009-07-20

**Authors:** Nicholas D Gilson, Anna Puig-Ribera, Jim McKenna, Wendy J Brown, Nicola W Burton, Carlton B Cooke

**Affiliations:** 1 School of Human Movement Studies, Faculty of Health Sciences, The University of Queensland, Australia; 2 Llicenciatura de Ciències de l'Activitat Física i l'Esport, Departament de Ciències i Ciències Socials, Universitat de Vic, Catalonia, Spain; 3 Carnegie Research Institute, Faculty of Sport and Education, Leeds Metropolitan University, Leeds, Yorkshire, UK

## Abstract

**Background:**

Interventions designed to increase workplace physical activity may not automatically reduce high volumes of sitting, a behaviour independently linked to chronic diseases such as obesity and type II diabetes. This study compared the impact two different walking strategies had on step counts and reported sitting times.

**Methods:**

Participants were white-collar university employees (n = 179; age 41.3 ± 10.1 years; 141 women), who volunteered and undertook a standardised ten-week intervention at three sites. Pre-intervention step counts (Yamax SW-200) and self-reported sitting times were measured over five consecutive workdays. Using pre-intervention step counts, employees at each site were randomly allocated to a control group (n = 60; maintain normal behaviour), a route-based walking group (n = 60; at least 10 minutes sustained walking each workday) or an incidental walking group (n = 59; walking in workday tasks). Workday step counts and reported sitting times were re-assessed at the beginning, mid- and endpoint of intervention and group mean± SD steps/day and reported sitting times for pre-intervention and intervention measurement points compared using a mixed factorial ANOVA; paired sample-t-tests were used for follow-up, simple effect analyses.

**Results:**

A significant interactive effect (F = 3.5; p < 0.003) was found between group and step counts. Daily steps for controls decreased over the intervention period (-391 steps/day) and increased for route (968 steps/day; *t *= 3.9, p < 0.000) and incidental (699 steps/day; *t *= 2.5, p < 0.014) groups. There were no significant changes for reported sitting times, but average values did decrease relative to the control (routes group = 7 minutes/day; incidental group = 15 minutes/day). Reductions were most evident for the incidental group in the first week of intervention, where reported sitting decreased by an average of 21 minutes/day (t = 1.9; p < 0.057).

**Conclusion:**

Compared to controls, both route and incidental walking increased physical activity in white-collar employees. Our data suggests that workplace walking, particularly through incidental movement, also has the potential to decrease employee sitting times, but there is a need for on-going research using concurrent and objective measures of sitting, standing and walking.

## Background

Walking is a practical and accessible form of physical activity through which people can develop and maintain their health [1]. Small step increments have been suggested to lower risk for chronic disease in sedentary and low active adults [2]. Experts also agree that accumulating at least 10,000 daily steps represents a minimal public health goal and that 3000–4000 of these steps should be achieved through bouts of brisk walking (3–4 mph), sustained for 10 minutes or more [3].

Two recent systematic reviews identify the workplace as an effective setting through which walking can be encouraged [4,5]. Evidence also shows that employees can achieve a range of benefits through worksite walking initiatives. These include reductions in hypertension [6] and high waist circumference [7] and improvements in perceptions of wellbeing, job satisfaction and productivity [8]. Studies also suggest that employees who spend more time walking tend to be less sedentary – typically defined as sitting [9,10]. For many people, the majority of sitting time occurs at work [11] and high volumes of sitting have been cited as an independent risk factor for conditions such as type II diabetes and obesity [12]. These data have led for calls to develop policy guidelines aimed at reducing sedentary behavior through frequent breaks in prolonged sitting [13,14]. Hamilton et al [13] make the important point that guidelines should complement, rather than replace those existing for physical activity, while Healy et al [14] have highlighted that accelerometer counts of 100, over at least a minute's duration, can be viewed as appropriate break-related criteria.

It seems reasonable to assume that increased standing and walking reduces time spent sitting. The results of a quasi-experimental, whole community intervention (n = 866) which promoted a 10,000 steps message supports this notion, showing an average reduction of 18 minutes daily reported sitting time, with increased step counts over a one year period [15]. However, the capacity of worksite walking programs to influence both behaviors remains unclear. Recent commentary in the field questions the notion that physical activity and sedentary behaviour are "opposite sides of the same coin" [16]. Evidence specific to the workplace also tends to be cross-sectional [9,10], and studies generally focus on physical activity as an outcome variable, while overlooking sedentary behavior [17-19].

In light of this, and in recognition of emerging debate around a) the physical activity/sedentary behaviour dichotomy [16], and b) sedentary behavior as an independent risk factor for chronic disease [12], this paper examined the impact of two workplace walking strategies on step counts and reported sitting times. The first strategy involved promoting planned route-based walking and the second, incidental walking in work-related tasks. We hypothesized that these different approaches, while increasing step counts, would have divergent effects on sedentary behaviour, with incidental walking having a greater effect on reduced reported sitting times.

## Methods

### Participants

Employees who took part in the study were white-collar university staff from the UK (n = 64; age = 41.4 ± 10.4 years; 58 women), Australia (n = 70; age = 43.1 ± 10.8 years; 54 women) and Spain (n = 80; age 39.1 ± 9.7 years; 58 women). These employees completed a collaborative workplace walking project, which in total ran during the months September-to-March 2005–06 (UK) and 2006–07 (Australia and Spain). Institutions involved were major regional universities, represented by a lead investigator, who had expressed an interest in running an employee intervention at their respective university as part of an evolving, international project. Informed consent was provided by all participants and study protocol approved by the ethics committee of each university.

### Measures

Trained and experienced researchers implemented the same standardized research protocol at each site. Personal information (age, gender and contact details), and body mass index (kg/m^2^; calculated from researcher assessed height and body mass), were recorded pre-intervention (September at each site). Step counts were also reported (waking-to-bedtime), using a diary and an unsealed pedometer (Yamax SW-200) for five consecutive workdays (Monday-Friday) – employees kept and used their pedometers throughout the duration of the project. Workday sitting times (hours and minutes) were measured using a logbook with question format based on other logbook research [9]. We adapted these questions to specifically refer to the context of the workplace and, to aid recall, employees reported the number of hours and minutes spent sitting, at the end of each morning (including lunch break) and afternoon work period. There is a lack of validity and reliability data for assessing sitting through logbooks, yet they are less dependent on long-term recall, and therefore in all probability more accurate, while providing a practical, less expensive alternative to objective monitors [20].

#### Protocols and Interventions

Pre-intervention workday step counts and block stratification were used to randomly and equally assign participants at each site to a waiting list control or one of two intervention groups. Control group participants were asked to maintain their normal behavior over a ten-week period (October-December at each site), while intervention participants were asked to increase their step counts. Employees in the first intervention group were directed to achieve this through brisk, sustained, route-based walking during work breaks. The second intervention group was asked to engage in incidental walking and accumulate step counts during working tasks – this strategy targeted walking and talking to colleagues, rather than sending emails or making telephone calls, and standing and walking in meetings, instead of sitting at desks. Importantly, participants in all groups were instructed not to engage in additional physical activities beyond those usually undertaken and – for route and incidental groups – the walking strategies encouraged in the workplace as part of intervention. Employees were asked to report additional activities or unusual workdays in their pedometer diaries.

#### Support Strategies

Intervention strategies used an ecological approach to facilitate and support changes in walking and sitting behavior. Employees were asked to use their pedometer as a motivational and self-regulatory tool – as a general guide, those above 10,000 daily steps at pre-intervention were encouraged to maintain this level of workday walking and add additional steps where possible. We encouraged relative and progressive, weekly increases of at least 1000 steps/day in those below a 10,000 steps threshold – this approach was based on guidelines for minimal walking bouts [3], allied with practical considerations around workday time demands.

Detailed instructions on goals and strategies to effect change were provided prior to intervention and then reinforced through weekly group emails – these contained motivational messages and reminders for control employees to maintain normal behaviour. Suggested campus walks, supported by maps, times (10-to-45 minutes) and step counts were provided for the routes group. Incidental employees were encouraged to exploit their office physical environments as a means of increasing steps (i.e. using the toilet at the far end of their building, rather than next door to their office) and to engage managers in providing opportunities for walking in work-tasks (i.e. absenteeism from desks while delivering messages to colleagues).

#### Measurement Points and Analyses

Workday step counts and reported sitting times were re-assessed for five consecutive days at the beginning (week one), mid- (week five) and endpoint (week ten) of intervention. Three intervention measurement points were selected in an effort not to overload participants with continuous recording, while at the same time enabling assessment of impact at equidistant time points – measurement at week one was specifically included, thereby allowing insight into how acute effects may translate into mid and endpoint assessments.

Pre- and intervention data were inputted into SPSS (Version 15.0; SPSS Inc, Chicago, IL, 2006) by lead researchers at each university, who were instructed not to enter data from workdays that employees reported as unusual or not normal (e.g. when they were sick, on holiday or engaged in physical activities which were not part of their normal routine or intervention goals). Individual sites provided feedback to employees following intervention completion (January to March at each site); on request, control employees were also given access to intervention materials during this period.

Lead researchers forwarded SPSS files electronically to a coordinating researcher where data were pooled and treated in the following ways. Participants with missing step count and/or reported sitting data at pre-intervention were removed, as were those with missing data for two or more intervention measurement points (i.e. step counts for weeks one and five) – those with one missing measurement point were included in analyses, with imputation of the missing data point as an average of the remaining two measurement points. Using recently published guidelines [3], employees were classified as "highly active (<12,500 steps/day), active (10,000 – 12,499 steps/day), somewhat active (7,500–9,999 steps/day), low active (5,000–7499 steps/day) or inactive (<5000 steps/day)" – these categories were assigned for both pre-intervention and intervention (average of week one, five and ten) step counts. The last of these categories we termed "inactive", rather than the previously used term "sedentary" [3], in order to avoid confusion with sedentary behavior defined as sitting.

MANOVA was used to analyze interactive effects between gender, site and group, relative to age and BMI. Step count categories were used to contrast the magnitude of change in walking (intervention average – pre-intervention step counts) relative to daily steps at pre-intervention. Steps/day and daily reported sitting times were compared using a mixed factorial ANOVA with timeline (pre; week one; week five; week ten) as the within participant factor and gender (male; female), site (UK; Australia; Spain) and group (control; routes; incidental) as the between participant factors. Group by timeline interactions were used to identify significant intervention effects, with paired sample-t-tests for follow-up, simple effect analyses. Alpha was set at p < 0.05.

## Results

### Pre-Intervention Characteristics

From a potential sample size of n = 214, 16% of participants (n = 35) had missing data at pre-intervention or two or more intervention measurement points – these data were removed prior to analyses, resulting in a final sample size of n = 179. Table 1 shows the relative contribution of each site to control, route and incidental groups within this final sample, along with the gender, age and BMI characteristics of each group. Numbers for groups were evenly distributed within the sample and their characteristics similar, with analyses showing no significant gender or site interactions for group age, BMI, daily steps or reported sitting times.

**Table 1:** Site contributions to groups and sample characteristics, by site and group, for participants with complete data.

Group	Site	n	Women/Men	Age (years)	BMI (kg/m^2^)
Control	UK	21	20/1	40.5 ± 11.0	25.4 ± 3.8
	Australia	16	12/4	44.5 ± 12.1	24.3 ± 5.1
	Spain	23	17/6	38.5 ± 11.1	23.0 ± 2.6
	Total	60	49/11	40.8 ± 11.4	24.2 ± 3.8

Routes	UK	21	19/2	43.8 ± 10.2	25.2 ± 4.1
	Australia	19	13/6	43.3 ± 10.0	26.7 ± 4.4
	Spain	20	13/7	39.4 ± 7.0	23.5 ± 2.8
	Total	60	45/15	42.1 ± 9.2	25.1 ± 4.0

Incidental	UK	21	18/3	39.8 ± 10.4	25.1 ± 3.4
	Australia	14	11/3	43.2 ± 10.3	28.1 ± 6.0
	Spain	24	18/6	40.8 ± 8.9	24.0 ± 3.1
	Total	59	4712	41.0 ± 9.7	25.4 ± 4.3

On average, pre-intervention data showed that employees sat for 337 ± 114 minutes/day (or 5.37 hours). They were also "somewhat" active [3] recording a mean daily step count of 9,360 ± 3165 daily steps. 35% of employees fell into this category, 26% were "low active" and 5% were "inactive". Of those remaining 18% of employees were "active" and 16% were "highly active".

### Intervention Effects

**Figure 1:**
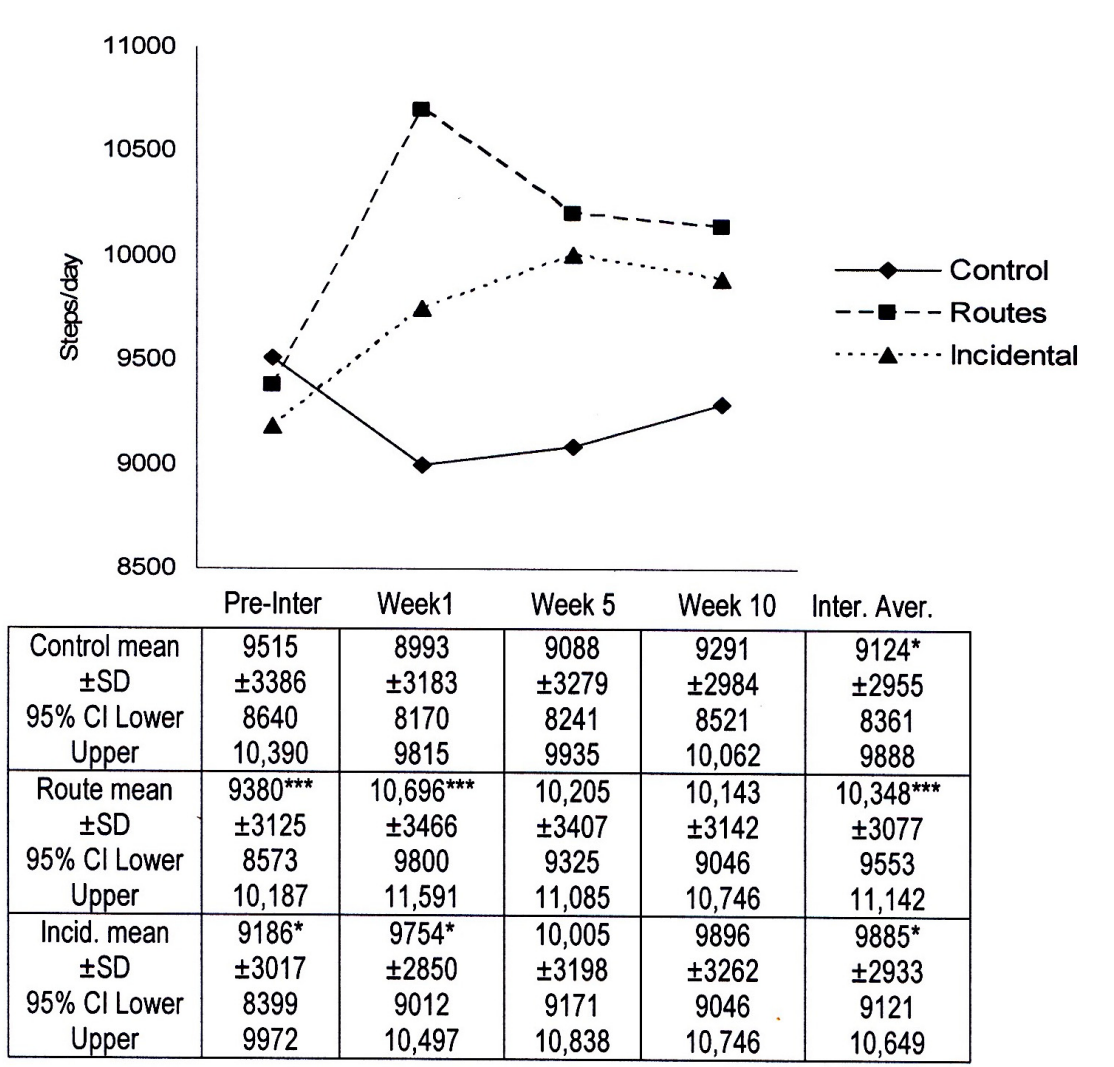
Mean ± SD and 95% confidence intervals for pre- and intervention sleep steps/day (*p < 0.05; **p < 0.001).

Figure 1 illustrates daily step profiles for groups across the pre- and intervention timeline. A significant interactive effect (F = 3.5; p < 0.003) was found between group and timeline for step counts; follow-up simple effects analyses showed significant differences for routes (pre-intervention vs week one: *t *= 4.7; p < 0.000) and incidental (pre-intervention vs week one: *t *= 2.1; p < 0.038) groups. An overall comparison of pre- against intervention average step count data (Figure 1) showed a non-significant decrease in the control group (-391 steps/day *t *= 1.76; p < 0.08) and significant increases in both the routes (968 steps/day; *t *= 3.9; p < 0.000) and the incidental (699 steps/day; *t *= 2.5; p < 0.014) group. Data viewed across step count classifications [3], showed that the magnitude of step count change progressively increased relative to pre-intervention step count classifications. "Inactive" (<5000 daily steps) routes and incidental employees demonstrated the largest change in workday walking; comparisons with "highly active" [>12,500 daily steps] employees evidencing mean differences of 2,312 and 2,166 steps/day respectively.

**Figure 2:**
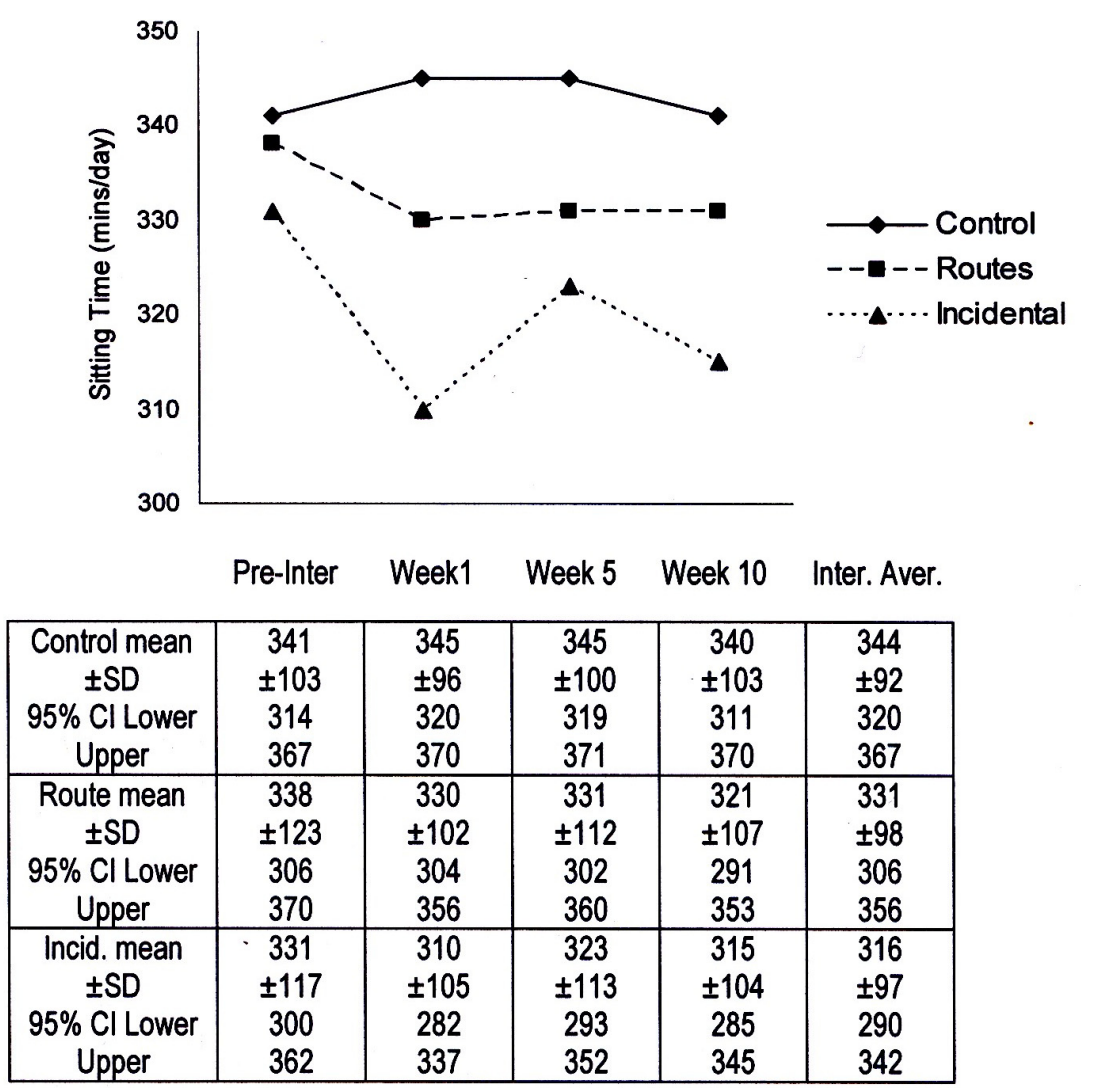
**Mean ± SD and 95% confidence intervals for pre- and intervention sitting times (mins/day)**.

Figure 2 shows daily reported sitting times for groups across the pre- and intervention timeline. No significant interactive effect was observed between group and timeline for these sitting times. However, inspection of the data shown in Figure 2 suggested a tendency for reported sitting times to decrease in the intervention groups and increase minimally in the control group. A trend for reduced sedentary behavior was most noticeable in the incidental group at week one of the intervention where, in comparison to pre-intervention values, reported sitting times decreased by an average of 21 minutes/day (t = 1.9; p < 0.057).

## Discussion

### Pre-Intervention Step Counts and Reported Sitting Times

Typical pre-intervention step counts for the sample (9,360 ± 3165 daily steps) were similar to values reported in New Zealand university employees (n = 88; 9498–9702 steps/day) [21]. The majority of our participants fell below the "active" 10,000 daily step threshold, with the sample containing a minority who were either "active" or "highly active". Although comparisons should be made with caution, given variations in occupational roles, our sample had a higher level of work-related sedentary behavior (5.37 hours/day of sitting) than Dutch educational sector workers [11] (n = 555) and Australian white-collar workers [22] (n = 409), who sat for around 1.30 and 3.30 hours/day respectively.

### The Impact of Route and Incidental Walking on Step Counts

Both groups significantly increased walking compared to a control. However, variability of step count change was high within intervention groups, suggesting that samples contained employees for whom our intervention worked, as well as those for whom change was not so evident. Employees classified as "inactive" at pre-intervention showed the highest magnitude of change in step counts and this supports the view advocated by other research, that walking interventions are most efficacious when directed at those most in need [4].

### Walking Interventions and Impact on Reported Sitting

Neither intervention group significantly decreased reported sitting times, suggesting that step count increases did not fully translate to reductions in sedentary behaviour. Questions therefore remain around the extent to which our walking interventions encouraged decreases in reported sitting.

This said reported sitting times remained relatively stable for control employees across ten weeks. In contrast, intervention participants demonstrated some decreases and qualitative data has indicated that a sub-sample of our intervention employees effectively engaged with their respective walking programs at the expense of reported sitting [8]. For routes-based employees, this involved sustained walking of at least ten minutes duration, during work breaks that may have otherwise been spent sitting. Overall decreases in reported sitting time (seven minutes/day) and increases in step counts (968 steps/day or nine-ten minutes/day) were comparable, if 100 steps are considered to equate to around one minute of walking for the average person [23].

Decreases in reported sitting times were most noticeable for the incidental group across ten weeks and this finding provides partial support for the hypothesis that incidental walking had the greatest impact on sedentary behavior. However, unlike the routes group, analyses showed decreases in reported sitting (15 minutes/day) were less comparable with increases in walking (699 steps/day or 6–7 minutes/day). One explanation for this disparity is that incidental group employees were encouraged to stand, as well as walk in work-tasks, so that time spent sitting reduced without concomitant increases in step counts. Increases in standing can only be inferred from our data, but reports highlight the contributions standing makes to increased energy expenditure, reduced weight gain and metabolic markers for disease such as triglycerides and plasma glucose levels [13,14].

### Study Limitations and Strengths

A Lack of objective and concurrent data on sitting, standing and walking was the main limitation of this study. Since the design and inception of our multi-site project in 2005, devices capable of accurately measuring sit-stand-walk continuum have been identified [24] and while costs may be prohibitive for community-level interventions, they should be used in future, settings-based contexts. Other limitations included group gender distributions dominated by women and convenience samples drawn from the higher education sector; these factors limit the generalization of findings to men and other workplaces. Our convenience samples were also relatively small which, in combination with lost data, may have impacted statistical power and reduced the ability to detect significant changes in reported sitting times. Detection of significant reductions in sitting time of ten minutes/day (with a standard deviation twice this size at 90% power), would have required minimum sample sizes of 84 employees per comparison group.

Study strengths included a randomized controlled design and the use of different walking strategies, applied to workplace physical activity and sedentary behavior; to our knowledge, this study is the first randomized control trial to investigate the impact different types of workplace walking interventions have on employee step counts and reported sitting times. Multi-site data were also valuable and the lack of significant interactions between universities inferred a degree of intervention success across different sites. Finally, timeline analyses provided useful data around the pattern and variability of change across the intervention period. Intervention groups demonstrated changes over ten weeks, yet the magnitude of this change was most apparent in week one, particularly for incidental employees, where decreased sitting approached significance. Future research will need to investigate why acute effects taper from this point and how researchers and practitioners can effectively sustain increased walking and decreased sitting in the workplace.

## Conclusion

This study investigated the impact different types of workplace walking strategies had on university employee step counts and sitting times. Compared to a control, both route and incidental groups significantly increased physical activity – this was most apparent for employees in lower active categories at pre-intervention. Decreases in sitting were also found relative to a control, particularly for incidental employees, but these decreases were non-significant and acute.

Our findings contribute to a developing field of enquiry and suggest that workplace walking strategies, particularly those that focus on incidental movement, impact step counts and also have the potential to impact sitting. Yet questions remain around sustainability and the extent to which increases in walking translate to reductions in sedentary behavior. This is due, in large part, to the need for concurrent and objective measurement of sitting, standing and walking.

## Competing interests

The authors declare that they have no competing interests.

## Authors' contributions

NG led the project, contributed to study design and conception, acquisition of data and overall analyses, drafted the manuscript and made final revisions for submission. APR, JM, WJB, NWB and CBC contributed to the design and conception of the study, were involved in the acquisition of data and critically revised the manuscript draft for final approval and submission. All authors read and approved the final manuscript.
